# Research progress on macrophage polarization during osteoarthritis disease progression: a review

**DOI:** 10.1186/s13018-024-05052-9

**Published:** 2024-09-28

**Authors:** Xiangzhi Yin, Quan Wang, Yijie Tang, Tianrui Wang, Yingze Zhang, Tengbo Yu

**Affiliations:** 1 Department of Orthopaedics, Affiliated Hospital of Qingdao University, Qingdao, 266000 China; 2 Department of Radiation Oncology, The Affiliated Hospital of Qingdao University, Qingdao, 266005 China; 3 Department of Orthopaedics, Qingdao Municipal Hospital, Qingdao, 266011 China

**Keywords:** Osteoarthritis, Macrophages, Polarization, Synovitis, Cartilage injury, Review

## Abstract

Primary osteoarthritis (OA) is a prevalent degenerative joint disease that mostly affects the knee joint. It is a condition that occurs around the world. Because of the aging population and the increase in obesity prevalence, the incidence of primary OA is increasing each year. Joint replacement can completely subside the pain and minimize movement disorders caused by advanced OA, while nonsteroidal drugs and injection of sodium hyaluronate into the joint cavity can only partially relieve the pain; hence, it is critical to search for new methods to treat OA. Increasing lines of evidence show that primary OA is a chronic inflammatory disorder, with synovial inflammation as the main characteristic. Macrophages, as one of the immune cells, can be polarized to produce M1 (proinflammatory) and M2 (anti-inflammatory) types during synovial inflammation in OA. Following polarization, macrophages do not come in direct contact with chondrocytes; however, they affect chondrocyte metabolism through paracrine production of a significant quantity of inflammatory cytokines, matrix metalloproteinases, and growth factors and thus participate in inducing joint pain, cartilage injury, angiogenesis, and osteophyte formation. The main pathways that influence the polarization of macrophages are the Toll-like receptor and NF-κB pathways. The study of how macrophage polarization affects OA disease progression has gradually become one of the approaches to prevent and treat OA. Experimental studies have found that the treatment of macrophage polarization in primary OA can effectively relieve synovial inflammation and reduce cartilage damage. The present article summarizes the influence of inflammatory factors secreted by macrophages after polarization on OA disease progression, the main signaling pathways that induce macrophage differentiation, and the role of different polarized types of macrophages in OA; thus, providing a reference for preventing and treating primary OA.

## Introduction

Primary OA is a common chronic disease with pain and loss of motor function as the characteristic disease manifestations [1]. Previous studies have shown that with the aging of population and the high obesity rate, OA has become a major musculoskeletal disease globally that affects the daily activities of the elderly population [2, 3]. OA affects women more frequently than males in those over 50. In OA patients with age ≥ 65 years, OA occurs in the hand, knee, and hip in 60%, 33%, and 5% of the patients, respectively; however, patients with OA in the hip and knee experience a higher degree of pain and disability [4]. Presently, clinical treatment of OA mainly includes nondrug therapy, drug therapy, and surgical therapy [1, 5, 6]. Knee OA can be treated non-operatively through diet management, appropriate exercise, and drugs such as non-steroidal anti-inflammatory drugs and intraarticular hyaluronic acid (IAHA) [7, 8]. For the overall treatment of hip OA, non-drug therapy is still the strongest recommendation due to the lack of drug clinical research data, while for the treatment of hand OA, drug therapy is mainly used, especially non-steroidal anti-inflammatory drugs or selective COX-2 inhibitors [7, 9]. Since the intervention for treating the disease mainly occurs in the late stage of OA progression, the therapeutic effect of nondrug treatments such as platelet-rich plasma (PRP), nonsteroidal anti-inflammatory drugs, and opioids is limited. Except for hip and knee replacement, which can treat end-stage diseases, there is no other approach that can delay the disease progression and the irreversible damage of cartilage [2, 9, 10]. Therefore, it is urgent and important to develop new methods for the treatment of OA.

Previously, primary OA was thought to be a degenerative disease; nevertheless, in the past decade, increasing lines of evidence show that OA is a multifactor disease, and low-grade, chronic synovial inflammation plays an important role in OA. As a separate risk element for new knee OA, synovitis is caused by multiple aspects, including not only cartilage and meniscus injury but also ligament injury and crystal deposition [11]. Modern imaging techniques such as magnetic resonance imaging and ultrasound examination have confirmed the role of synovitis in primary OA pain and changes in bone and cartilage structure; however, the degree of synovitis needs to reach a certain threshold to affect OA disease progression [11, 12]. Synovial inflammation is mainly mediated by immune cells, particularly macrophages, which are the primary participants in chronic synovial inflammation, osteophyte formation, joint pain, subchondral bone remodeling, and cartilage injury, and the extent of macrophage activation is also correlated with OA severity [13–17].

**Fig. 1:**
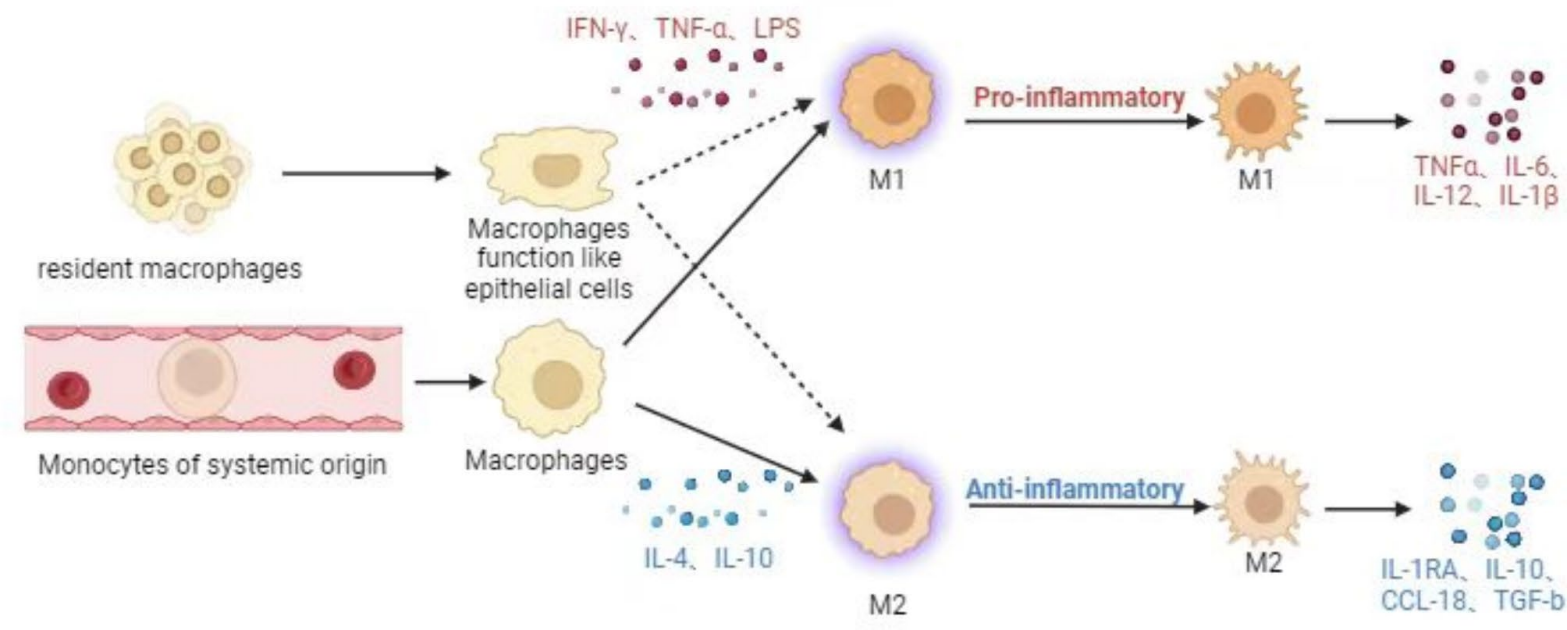
Origin and polarization of macrophages

Macrophages are commonly categorized into two types: classically activated M1 type and alternately activated M2 type. Based on the expression of CD markers on the cell surface, M1 and M2 macrophages are classified as CD11c + CD206- and CD11c-CD206+, respectively. M1 macrophages are activated by environmental factors like interferon-γ (IFN-γ), tumor necrosis factor-α (TNF-α), and lipopolysaccharide (LPS), and they secrete proinflammatory cytokines such as IL-1, IL-6, and low levels of IL-10. M2 macrophages have anti-inflammatory properties and tissue repair function [18, 19] (Fig. 1). By generating cytokines including TGF-β, IL-10, CCL-18, and IL-1RA, these macrophages contribute to the reduction of inflammation(Fig. 1). The spatial and temporal distribution of M1 and M2 macrophages are essential for the accurate control of inflammation and the regeneration of tissue. During OA disease progression, the ratio of M1/M2 macrophages constantly changes, and the imbalance between proinflammatory and anti-inflammatory macrophages is one of the causes of low-grade chronic inflammation [20]. It is, however, interesting to note that the simple depletion of macrophages does not delay primary OA progression; in contrast, it aggravates synovitis and increases the production of inflammatory cytokines. Therefore, more targeted approaches for different polarized macrophage subsets are required for primary OA prevention and treatment [14, 21]. Previous studies have shown that macrophages are involved in skeletal muscle and tendon repair [22, 23]. However, targeted therapies on how to slow down the primary OA process by regulating macrophage polarization are yet to be developed. This study focuses on the function of synovial macrophages and their subsets in OA and the paracrine effects they have on chondrocytes, adipose tissue, and neovascularization. We also highlight the signaling pathways that regulate macrophage polarization and provide an overview of the pertinent mediators released by macrophages that are important in the progression of OA. We aimed to find a new target to treat or delay primary OA disease by studying macrophages and their polarization process in primary OA.

## Polarization of macrophages in OA

### Synovium and macrophage polarization

**Fig. 2:**
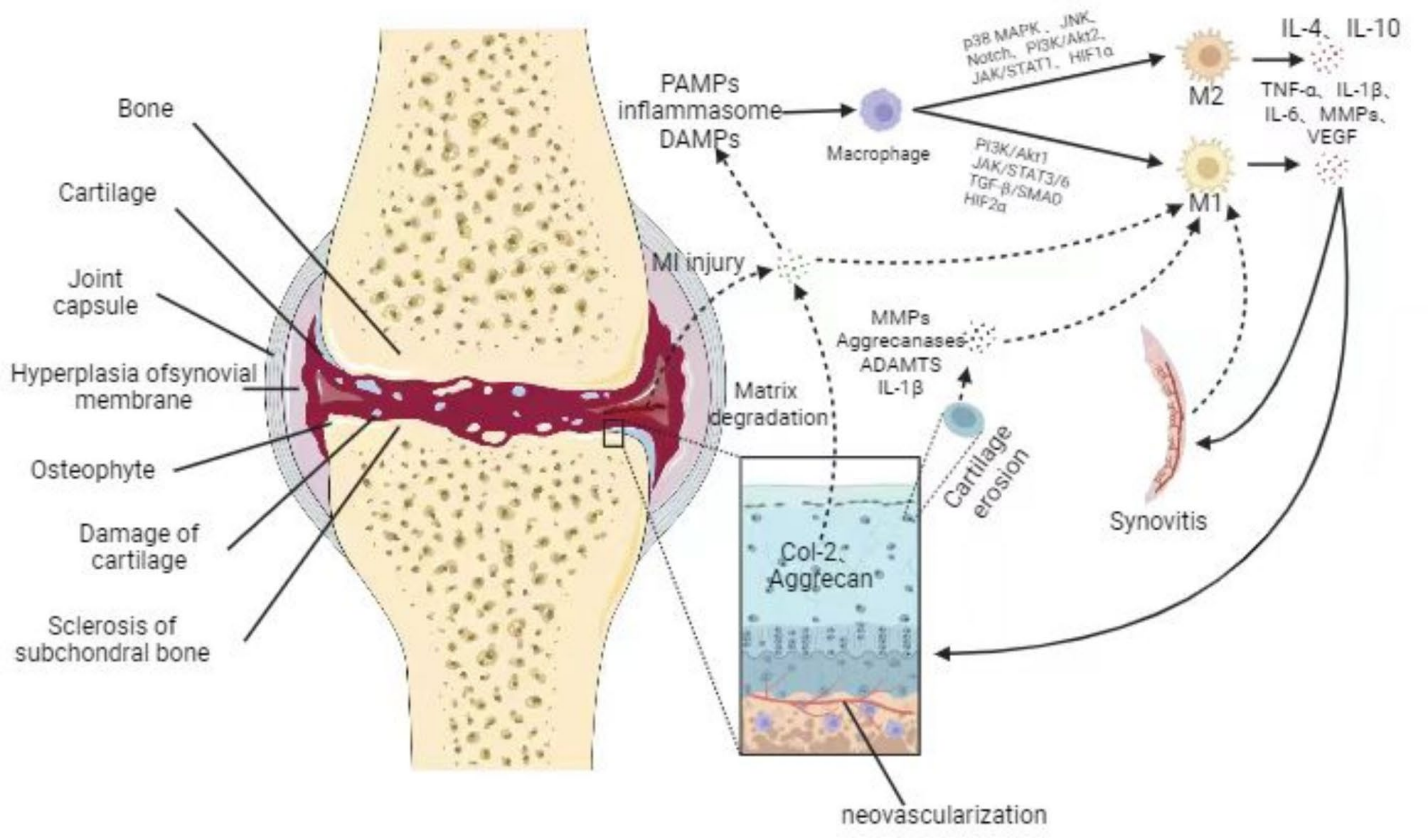
Activation and signal transmission of TLR signal transduction

**Fig. 3:**
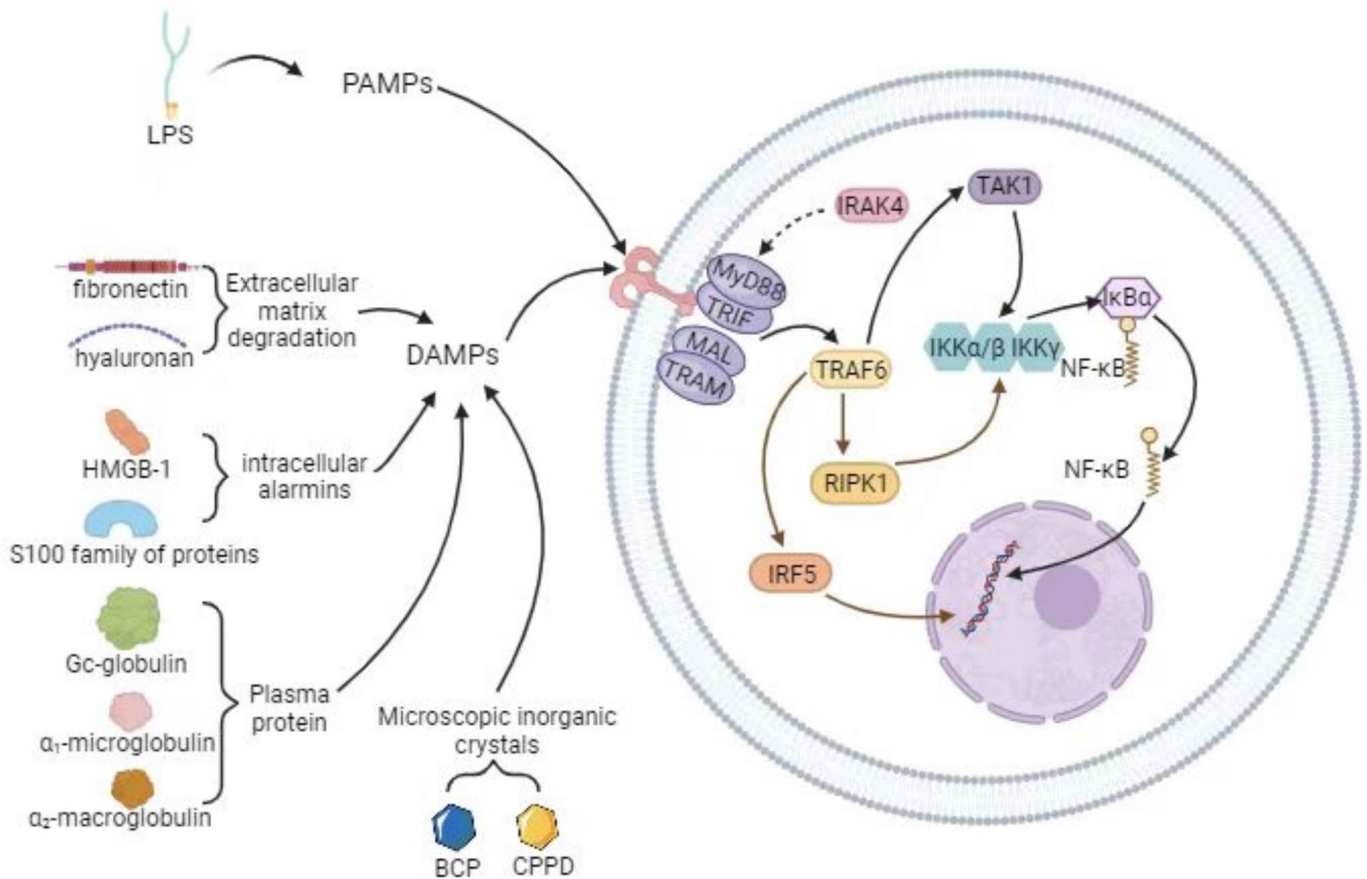
Cartilage degradation and the generation of macrophage pro- or anti-inflammatory factors

Human joint synovium, as a macrophage-rich area in joints, is composed of two layers. The first layer is an intimal lining layer containing macrophages and fibroblast-like synovium cells with secretory function. The second layer, known as the synovial sublining layer, is mostly made up of blood vessels and fibrous connective tissues. It contains very few macrophages and lymphocytes [24, 25]. A central feature of primary OA is activation of the innate immune system [26–29]. This is different from the characteristics of rheumatoid arthritis, which repeatedly and continuously activates the innate and acquired immune system, leading to immune tolerance, autoantibody production and excessive production of inflammatory cytokines in the later stage of the disease [26–29]. The investigation of tissue-resident macrophages in mouse primary OA revealed that resident macrophages in OA can act as a barrier similar to epithelial cells and shield proinflammatory signals through tight junctions between cells to limit early inflammatory responses [18] (Fig. 1). Human subject tissue-resident macrophages can be subdivided into CD11c-CD206-, CD11c + CD206- (M1), CD11c-CD206+ (M2), and CD11c + CD206 + macrophages according to CD markers [19, 30]. Nonetheless, some research has demonstrated that human peripheral blood monocyte-derived macrophages (MDMS), which are classified according to CD markers as CD86-CD206-, CD86 + CD206-, CD86-CD206+, and CD86 + CD206 + macrophages, are the primary agents in the inflammatory aspects of the illness [19, 30]. Among them, CD86 + CD206- and CD86-CD206 + macrophages had similar effects with M1 and M2 macrophages, respectively, and the transcription factors Pu.1, CEBP-α, CEBP-β, and Jun can control the differentiation of systemic monocytes into proinflammatory synovial macrophages [19, 30, 31]. At the beginning of inflammation, hyaluronic acid and fibronectin released from the extracellular matrix and intracellular proteins from stressed, damaged, or necrotic cells serve as endogenous damage-associated molecular patterns (DAMPs) through the Toll-like receptor (TLR) pathway (Fig. 2). Finally, signaling cascades activate transcription factors such as interferon regulatory factors (IRFs), AP-1, and nuclear factors (NF-kB), thereby producing chemokines (such as IL-5 and CCL1) and cytokines (such as IL-6, IL-2, and TNF). Additionally, the damaged meniscus and ligaments also produce inflammatory signals, and these inflammatory signals and chemokines can recruit and activate MDMS, thereby exacerbating the inflammatory response [32–34] (Fig. 3). Proinflammatory cytokines and stroma-degrading enzymes (matrix metalloproteinase(MMP)) generated during the inflammatory response diffuse to cartilage through the synovial fluid. At the end of this process, the cartilage gradually degenerates, causing more inflammatory signals to be produced, maintaining and exacerbating the illness [35] (Fig. 3).

Macrophages can also be activated by the inflammasome. Pyrin domain 3 (NLRP3) is an intracellular protein complex that participates in the activation of IL-1β and IL-18 by splitting pro-caspase-1 into caspase-1, thereby promoting inflammatory cytokine production. Calcium phosphate crystals commonly found in the joint fluid of OA patients, together with uric acid, also participate in the proinflammatory process of the NLRP3 inflammasome [36]. However, it remains unclear whether the mechanism of caspase-1 activation depends on the activation of this inflammasome [37]. Zhou et al. used extracellular vesicles (EVs) of human umbilical cord mesenchymal stem cells to inhibit the expression of methyltransferase-like protein 3 (METTL3), diminish the m6A expression level of NLRP3 in macrophages, and inhibit the activity of NLRP3 in a mouse OA model, which exerted anti-inflammatory effects [38].

These studies suggest that synovial inflammation is an important part of OA progression and emphasize the role of polarized macrophages in promoting inflammation and cartilage damage. Therefore, targeting and regulating polarized macrophages appear to be an effective approach to prevent and treat OA(Table 1).

Articular macrophages can be classified into resident macrophages and MDM, among which resident macrophages can perform epithelial-like barrier function and intercellular connections can shield inflammatory signals during disease progression. Nonetheless a portion of resident macrophages can still differentiate into pro-inflammatory macrophages. The cells from both sources differentiate into the M1 phenotype under the stimulation of IFN-γ, TNF-α, and LPS and secrete proinflammatory cytokines such as TNF-α, IL-1, IL-6, and IL-10, which played the pro-inflammatory role of macrophages. Following stimulation by IL-4 and IL-10, they can differentiate into the M2 phenotype and secrete anti-inflammatory cytokines IL-1RA, IL-10, CCL-18, and TGF-β. M2 phenotype can secrete anti-inflammatory cytokines to play an anti-inflammatory role. MDM has a significant impact on how primary OA develops.

### Infrapatellar fat pad and macrophage polarization

The infrapatellar fat pad (IPFP) is a reservoir of adipose tissue in the knee joint, which can secrete inflammatory mediators that affect synovial and cartilage inflammation and participate in OA disease progression. IPFP is also another location where macrophages exist in large numbers in the joint [39]. Prior researches have confirmed that the quantity of macrophages in the IPFP of OA patients increased significantly, and the proportion of M1-like (CD11C+) macrophages and M2-like (CD206+) macrophages increased [40]. Macrophages exist in an intercellular matrix composed of collagen and elastic fibers. The activation of various growth factors, cytokines, and enzymes produced by macrophages can enhance osteophyte formation, aggravate or reduce chondrolysis through MMP activity, induce joint effusion through vasodilation, and affect subchondral bone metabolism [39]. Through an immunomic analysis of macrophage subsets in IPFP of patients with osteoarthritis (OA) in the knee, Patchanika et al. discovered that OA-related genes were expressed at higher levels in CD11c + CD206 + macrophages as opposed to CD11c + CD206-, CD11c-CD206+, and CD11c-CD206- macrophages [19]. This is consistent with the conclusion that CD11c + CD206 + macrophages in adipose tissue secrete more inflammatory cytokines [41]. Additionally, Patchanika et al. observed that after treating MDM with IPFP-conditioned medium, there was a decrease in the expression of CD86 + CD206 + and CD86-CD206 + macrophages and an increase in the expression of CD86 + CD206 + macrophages [19]. In stark contrast, MDM treated with synovial tissue culture medium showed an increase in CD86 + CD206- macrophage phenotype and a decrease in CD86 + CD206 + macrophage phenotype [19]. As the closest adipose tissue and macrophage reservoir to the joint cavity, IPEP can affect the progression of human OA disease by affecting macrophage polarization.

### Macrophage polarization and neovascularization

The generation of new blood vessels is often accompanied by the growth of sensory nerves, and the joint pain of OA patients may be associated with the neovascularization of the synovium and osteochondral junction. Synovial angiogenesis in OA patients is directly proportional to the histological grade of macrophage infiltration and inflammation, and the vascular density at the osteo-cartilage junction is associated with the change in OA, which might be related to the large production of provascular growth factors such as vascular endothelial growth factor (VEGF) and its inducer hypoxia-induced-factor-1α (HIF-1α) [42–44]. However, a notable finding is that M2 macrophages are less sensitive to endogenous antivascular growth factor IL-10, and TNF-β produced by M2 can regulate the formation of H-type blood vessels and enhance VEGF production in articular cartilage exografts; thus, it may play a more important role in synovial and subchondral bone angiogenesis [42]. Lowin et al. observed that androgen/estrogen transformation occurs in the tissues of OA patients, and estrogen can induce VEGF expression and promote the increase in the number of placental growth factor 1 (PlGF-1)-positive macrophages; the authors also found that the density of macrophages is positively correlated with the density of type IV collagen-positive blood vessels [45].

### Macrophage polarization and adipose tissue

Obesity is one of the most influential and changeable risk elements for primary OA, while healthy people show the predominance of M2 macrophages [46]. Obesity can induce the secretion of monocyte chemoattractant protein 1 (MCP-1) in adipose tissues and accelerate the recruitment and activation of MDMS. Following polarization, macrophages can release proinflammatory factors to interact with adipocytes through the paracrine mode [47]. The released MCP-1 can recruit and again activate macrophages; thus, this cycle is repeated to induce chronic inflammation in adipose tissues [47]. Saturated fatty acids released by adipose tissues can interact with the TLR4 complex to activate the NF-κB pathway. Leptin produced by adipose tissues can enable macrophages to express the M2 phenotype marker while secreting proinflammatory cytokines such as TNF-α, IL-6, IL-1β, IL-1ra, and IL-10 to regulate chondrocytes to produce MMPs and ADAMTS [48, 49] (Fig. 3). Adiponectin produced by adipose tissues can also inhibit M1 macrophage activation and upregulate M2 macrophage markers such as IL-10 and MgL-1 to play an anti-inflammatory role by downregulating TNF-α, IL-6, and MCP-1 [50]. By conducting experiments, Li confirmed that metformin can reduce leptin secretion in the adipose tissues of obese mice, thus reducing the level of proinflammatory factors and decreasing the infiltration and polarization of macrophages in adipose tissues [51](Table 1). However, it’s possible that conclusions from studies using mice won’t necessarily apply to people. Modulation of adipose-associated macrophages frequently has an impact on the body’s other macrophages [47]. Therefore, the specific role of adipose-associated macrophages is still unclear. More research is needed to determine whether inhibiting adipose tissue’s production of inflammatory cytokines and severing the macrophage-adipose tissue communication pathway can delay the progression of osteoarthritis and control macrophage reprogramming.

Articular cartilage degeneration, subchondral bone thickening, osteophyte production, meniscus degradation and variable degrees of synovial inflammation are typical features of primary OA. PAMPs, DAMPs, and the inflammasome constitute the main microenvironment for macrophage polarization. M1 phenotype is regulated by p38 MAPK, JNK, HIF-1α, and other pathways, while M2 phenotype is regulated by PI3K/Akt1, JAK/STAT3/6, and other pathways. These polarized macrophages secrete proinflammatory factors such as TNF-α, IL-1β, IL-6, and MMPs and anti-inflammatory factors such as IL-4 and IL-10. On the one hand, polarized M1 macrophage products can aggravate synovial inflammation and secrete VEGF, which promotes synovial neovascularization and osteochondral junction neovascularization. Moreover, the proinflammatory factors secreted by M1 macrophages can also promote articular cartilage damage and stimulate chondrocytes to secrete metabolic factors such as MMPs, ADAMTS, aggrecanase and IL-4D, which directly act on M1 macrophages to promote their further secretion of proinflammatory factors. On the other hand, injured meniscus and degraded cartilage extracellular matrix (ECM) can function as DMEM to stimulate macrophage polarization through several signaling mechanisms. These two aspects work together to aggravate the pain related to primary OA and to create a vicious cycle of cartilage degradation and synovial inflammation.

### Interaction between macrophage polarization and chondrocytes

Macrophage-induced inflammation has a significant impact on cartilage damage and subchondral bone remodeling during primary OA progression; however, the sequence of changes is uncertain. By conducting flow cytometry, Pippenger confirmed the presence of macrophages (CD45+/CD14+/CD68+) in the bone marrow tissues of subchondral trabecular bone in primary OA patients during disease progression; among these macrophages, CD68 + macrophages are associated with the new bone formation region [52, 53]. Polarized macrophages in human joints can produce higher levels of TNF-α, IL-1β, IL-6, and other cytokines through the paracrine mechanism, which can regulate the synthesis of the extracellular matrix of chondrocytes; this results in the degradation of matrix components such as aggrecan (ACAN) and type II collagen (COL2), and the degraded substances act as new DAMP. Cartilage injury in OA is a vicious cycle that involves stimulation of macrophage activation and exacerbation of synovial inflammation. Interestingly, the induction of TNF-α expression in cartilage can impair the production of protease agglutinase 1 (ADAMTS4); however, the production of agglutinase 2 (ADAMTS5) is not affected by TNF-α or IL-1β [54, 55]. Therefore, a better understanding of the crosstalk between inflammatory macrophages and chondrocytes may help to identify new therapeutic targets for the treatment of cartilage damage in primary OA.

Intercellular communication between activated macrophages and chondrocytes can also occur through EVs. Taku Ebata et al. collected EVs produced by activated macrophages and used them for the in vitro knee injection experiments of chondrocytes and mouse OA models(Table 1). The outcomes demonstrated a noteworthy increase in the expression of catabolic-related factors. RNA sequencing analysis disclosed that the upregulated genes were mainly linked to the apoptotic process and tumor necrosis factor signaling pathways. These findings suggest that activated macrophages induce the apoptosis of chondrocytes [56]. Other studies have shown that synovial macrophages can regulate the chondrogenic role of mesenchymal progenitor cells (MPCs) to affect cartilage injury in primary OA patients; however, the specific mechanisms remain unclear [57]. These results demonstrate that the signaling process between macrophages and chondrocytes is essential for primary OA cartilage injury. Recent studies have also revealed the effect of different polarized macrophages on OA chondrocytes. Zhang et al. successfully promoted the phenotypic transformation of macrophages from M1 to M2 by using exosomes derived from bone marrow mesenchymal stem cells in a rat model of OA [58]. In in vitro experiments, chondrocytes could maintain their growth characteristics and inhibit hypertrophy; moreover, the levels of the proinflammatory cytokines TNF-α, IL-6, and IL-1β were decreased, while the levels of the anti-inflammatory cytokine IL-10 were increased. In animal experiments, a decrease in osteophyte number reduced chondrocyte injury [58]. Thus, the polarization process of macrophages has a significant part in cartilage injury, and a better understanding of the paracrine and other signal exchange mechanisms between these two types of cells will help provide important clues for primary OA treatment.

Further studies have shown that pinocytosis of macrophages also has a significant impact on cartilage injury. The retention of the cytostatic capacity of M2 macrophages enables continuous elimination of apoptotic cells (ACs) and other DAMP, which is essential for maintaining the anti-inflammatory function [59]. Yao et al. reported that the secretion of growth arrest protein 6 (GAS6) decreased following the polarization of M1 macrophages in the synovial membrane of OA model mice, thereby resulting in reduced pinocytosis and phagocytosis of synovial macrophages to DAMP; moreover, the cell content released by accumulated ACs further activated the immune response, resulting in the release of TNF-α, IL-1β, IL-6, and other inflammatory factors [60]. The expression levels of chondrocyte proteins such as p16, p21, and MMP13 increased, and the injection of GAS6 into the articular cavity restored the macrophages’ phagocytic function and contributed to the maintenance of cartilage thickness [60].

These studies reveal that the complex signaling process between macrophages and chondrocytes is an important pathway that leads to cartilage injury and disease progression, and the paracrine production of inflammatory cytokines is the main aspect. However, most recent studies have focused on in vitro experiments, and there is no in vivo evidence to confirm the biological crosstalk and specific mechanisms of the association between macrophages and chondrocytes in the human body; this topic requires further research.

## Pathological changes of macrophages and OA

### Research based on radionuclides

Macrophage polarization is one of the markers of OA pathology. Kraus et al. used 99mTC-EC20 to label folate receptor-β (FR-β), which is highly expressed by macrophages, and 50 patients were examined by single photon emission computed tomography combined with high resolution computed tomography (SPECT-CT). The statistical analysis of the absorbed radioactive signal showed that the inflammation caused by macrophage polarization was closely related to the imaging severity of knee joint space stenosis and osteophytes. It is also believed that blocking the transformation of macrophages from M1 to M2 phenotype will inhibit the repair and healing process of OA [16]. Yang et al. used a radioactive probe cFLFLF-PEG-HYNIC-^99 m^Tc to specifically label formyl peptide receptor 1 (Fpr1) expressed by activated M1 macrophages in OA [61]. In a mouse model, the signal difference between the OA model group and the sham operation group was apparent in the early stage of the disease, and the signal intensity of the probe gradually decreased with disease progression However, it remains unclear whether this change is due to the differentiation of blood-derived monocytes into M1 macrophages or due to the reprogramming of M2 macrophages into M1 macrophages. M1 macrophages have a primary role in the disease’s early inflammatory response [61]. These studies confirm that macrophage polarization is involved in the entire process of OA disease progression and has a significant impact on the early stage of disease progression; thus, macrophage polarization can be used as an important reference for the early assessment of OA progression and disease prevention.

### Number and type of macrophages affect the pathological changes of OA

After determining the role of synovial inflammation in OA, the researchers such as Martin et al. assessed whether the number of macrophages influences the relationship between macrophages and OA [62, 63]. Bailey et al. injected clodronate enclosed in liposomes into the joint cavity to inhibit local joint macrophages or used the small molecule AP20187 to specifically inhibit macrophages in transgenic mice [64](Table 1). The results showed a rise in the proportion of M1 macrophages. However, it remains unclear whether this change is due to the differentiation of MDMS into M1 macrophages or due to the reprogramming of M2 macrophages into M1 macrophages [64]. Interestingly, the depletion of macrophages leads to severe synovial inflammation and aggravates OA [64]. Wu et al. also confirmed that macrophage depletion of mice with joint osteophytes significantly decreased, but did not reduce OA; however, it promoted the production of proinflammatory cytokines in serum and joints [21]. Additionally, the dexamethasone can promote synovial macrophage transformation into the M2 phenotype and thus has a chondroprotective effect; moreover, the intra-articular injection of triamcinolone into the knee joint of rats can increase the proportion of M2 macrophages and significantly reduce the formation of knee osteophytes [65, 66](Table 1). These studies confirmed that the number of polarized macrophages is closely associated with primary OA pain、synovitis and osteophyte formation; however, it remains to be determined whether M1 and M2 macrophages can be transformed in OA. Unfortunately, regulating macrophage reprogramming to treat OA is still in the experimental stage, and this aspect remains the focus of future research.

### Macrophages and pain in primary OA

Previous studies have also shown that macrophages are associated with abnormal pain in primary OA [67]. Cindy et al. injected hyaluronan hexadecylamide derivative and bone marrow-derived stem cells (MSCs) into the knee joint of an OA mouse model; the authors observed that pain reduction was associated with a reduction in macrophage numbers [68]. Macrophages can also regulate pain in the dorsal root ganglion (DRG) far from the injured site. Previous experiments have confirmed that sensory neurons innervating OA knee joints can promote macrophage transformation to M1 phenotype, thereby resulting in persistent joint pain; however, the pain is relieved after M2 macrophage injection into the DRG. These findings reveal the critical role of macrophages in maintaining primary OA pain and provide new directions for treating OA pain.

## Signaling pathway of macrophage polarization

Despite reprogramming macrophages is a promising therapeutic approach for treating inflammatory disorders, the processes controlling macrophage polarization are intricate [29]. The most typical one is mediated by TLRs. The TLRs are a group of membrane-associated pattern recognition receptors (PRRs) that react to pathogen-associated molecular patterns (PAMPs) like LPS in addition to recognizing endogenous DAMPs [69]. There are many types of DAMPs, including [1] extracellular matrix breakdown products; [2] intracellular proteins released from stressed and damaged cells, such as high mobility group protein B1 and S100 protein family; [3] plasma proteins such as GC-globulin, α1-microglobulin, and α2-macroglobulin; and [4] crystals present in the synovial fluid and tissues of joints, such as basic calcium phosphate (BCP) and calcium pyrophosphate dihydrate (CPPD) crystals [33] (Fig. 2). After the stimulation of PRRs by the signal, interleukin-1 receptor-associated kinase 4 (IRAK4) is recruited, binds to the TLR junction molecule MyD88, and undergoes phosphorylation, thereby prompting tumor necrosis factor receptor-associated factor 6 (TRAF6) to produce two modes of action, one of which involves binding to TGF-beta-activating kinase 1 (TAK-1) to generate signal transmission(Fig. 2). The activation of the trimer IκB kinase (IKK) complex composed of catalytic (IKKα and IKKβ) and regulatory (IKKγ) subunits leads to the ubiquitination of the NF-κB inhibitor (IκBα), release of NF-κB protein dimers for nuclear translocation, binding to specific targets in the nucleus, and initiation of the secretion of cytokines and chemokines such as TNF, IL-6, and MMP [70, 71] (Fig. 2). Another method involves recruiting receptor-interacting protein kinase 1 (RIPK1) for TRAF6, which transmits signals through the IKK complex or activates interferon regulatory factor 5 (IRF5) in the nucleus to bind to specific DNA elements [72] (Fig. 2). Stabler et al. established an in vitro model to demonstrate that chondroitin sulfate (CS) can influence the proinflammatory effect of NF-kB by inhibiting the downstream TLR pathway, wherein the classical pathway comprising heterodimers of p50/p65 and homodimers of p50 is the most affected one [73]. The Rel B specification (p50/Rel B) and C-Rel specification (p50/C-Rel) subunits are also affected [73]. Through in vitro experiments and a rat OA model, Xie et al. demonstrated that Alpha defensin-1 can induce the transformation of macrophages from M1 to M2 by affecting the TLR pathway [29](Table 1). In vitro Transwell co-culture experiments showed that the co-culture of M1 macrophages and chondrocytes increased the expression of OA-related proteases such as MMP-3, MMP-13, and ADAMTS5, which aggravated OA severity; in contrast, the use of Alpha defensin-1 reduced the level of the abovementioned proteases. This provides an alternative method to reprogram macrophages [29]. Existing studies have shown that TLR channels can suppress synovial inflammation and decrease the release of related inflammatory cytokines by M1 macrophages or promote M1 macrophage transformation into M2 macrophages. Because of the reversible state of macrophage polarization, the ideal approach to prevent these cells from inducing joint damage is to shift them from the M1 phenotype to the M2 phenotype.

In addition to the classical TLR pathway, there are other M1 phenotypic programming pathways, such as p38 MAPK, JNK, Notch, PI3K/Akt2, JAK/STAT1, and HIF-1α pathways, as well as major pathways that program transformation to the M2 phenotype, including PI3K/Akt1, JAK/STAT3/6, TGF-β/SMAD, and HIF-2α pathways [74] (Fig. 3). Yan et al. constructed a NAHA-CaP/siCA9 nanocore to inhibit the activation of p38 MAPK, NF-κB (p50/p65), and MyD88 signaling pathways, which reduced the production of proinflammatory factors and promoted the repolarization of macrophages from the M1 type to the M2 type [75]. Furthermore, pro-chondrogenic cytokine and matrix genes were shown to be upregulated in OA models in both rats and mice models [75]. Ji et al. used apigenin to inhibit M1 macrophage polarization and promote M2 macrophage polarization through the TRPM7-mTOR and MAPK pathways, alleviate the chondrocyte apoptosis and inflammatory response in the macrophage-chondrocyte co-culture system, and effectively slow down OA disease progression [76](Table 1). Sun et al. injected SHP099 into the knee joint to inhibit the TLR signaling of NF-κB and the PI3K-AKT signaling pathway during M1 polarization, which successfully alleviated joint synovitis and cartilage injury [77](Table 1). Nuclear factor-erythrocyte 2-associated factor-2 (Nrf2) is a transcription factor that encodes several antioxidant enzymes and has anti-inflammatory effects. Following its activation, Nrf2 can inhibit M1 polarization and promote M2 polarization through the TGF-β/SMAD, TLR/NF-κB, and JAK/STAT signaling pathways [69](Table 1). The polarization pathways of macrophages are numerous and may be reprogrammed by regulation. This reprogramming can then be utilized as a target to inhibit the progress of primary OA disease.

In general, the induction of differentiation and reprogramming of macrophages has increasingly become a research hotspot in OA treatment. Recent research also shows that the transformation of M1 macrophages into M2 macrophages can effectively inhibit the progression of OA inflammation, although M1 and M2 macrophages are only two extreme polarization conditions. Hence, studies of macrophage subpopulations may provide a more efficient approach to reprogram macrophage polarization [78–80].

Toll-like receptors (TLRS), composed of MyD88/TRIF or MAL/TRAM, receive stimulation from PAMPs and DAMPs. Among these, LPS is a representative of PAMPs, and DAMPs are made up of plasma proteins (GC-globulin, α1-microglobulin, α2-macroglobulin, etc.), intracellular products (hyaluronic acid and fibronectin, etc.) and microcrystals (BCP and CPPD). Following the binding of the ligand to TLR, IRAK4 is rapidly recruited and binds to MyD88 and other junction molecules to form a complex; this process prompts TRAF6 and TAK-1 to induce signal transmission, thereby activating the IKK complex composed of IKKα, IKKβ, and IKKγ. Following IκBα’s phosphorylation and ubiquitination as a result of IKKβ activity, NF-κB inhibitors are efficiently broken down by proteases, releasing NF-κB protein dimers. Simultaneously, the nuclear localization signal of NF-κB is obtained, enabling it to translocate to the nucleus and bind itself to particular locations within the target gene promoter region, thereby enhancing gene transcription. TRAF6 can also recruit RIPK1 while activating IRF5 into the nucleus to bind specific genes. The IKK-IκBα-Nf-κb axis can be activated by RIPK1 binding to the IKK complex, which can convey signals and impact the nuclear translocation of the NF-κB protein dimer.

### Cytokines secreted by polarized macrophages drive OA progression

Cytokines and related proteins secreted by synovial macrophages after polarization are the main factors that lead to OA inflammation; among these factors, interleukin-1β (IL-1β) and TNF-α produced by M1 macrophages are the main mediators that drive OA synovial inflammation and cartilage destruction [81]. This influence is multifaceted. Previous experiments have shown that IL-1β and TNF-α can induce increase in the levels of MMP1, MMP3, IL-6, and IL-8 and also increase NO release, which is accompanied by the loss of cartilage proteoglycan [82, 83]. Fei et al. used luteolin to inhibit IL-1β, which significantly reduced the level of inflammatory factors, reversed type II collagen degradation, and inhibited NF-κB phosphorylation [84]. Therefore, we can assume that cytokines secreted by macrophages will not only cause synovial inflammation but also lead to chondrocyte apoptosis and reduction in key synthetic components of the chondrocyte extracellular matrix, such as proteoglycan and type II collagen. In addition, compared to patients with early-stage OA, patients with end-stage OA show a reduction in the expression level of proinflammatory factors such as IL-1β, while the TNF-1β level changes with disease severity and can predict OA disease progression [85, 86].

Bone formation and remodeling are determined by the relationship between osteoclasts’ bone resorption and osteoblasts’ bone formation. IL-1β and TNF-α secreted by activated macrophages promote osteoclast maturation and affect bone metabolism by participating in the OPG/RANKL/RANK system composed of osteoprotegerina (OPG), NF-κB receptor activator ligand (RANKL), and NF-κB receptor activator (RANK). It also has adverse effects on the surrounding cartilage [87]. Macrophages have an impact on bone at every stage of the inflammatory response. During arthritis progression, fibroblast-like synovial cells (FLS) can promote macrophage polarization, which drives joint inflammation and bone destruction. During the bone repair process, vascular endothelial growth factor (VEGF) secreted by chondroblasts can recruit macrophages to participate in the process of bone remodeling and repair [88].

Macrophages also inhibit primary OA disease progression. Existing experimental studies have shown that IL-4 can stimulate macrophages to polarize into M2 type, promote the secretion of the anti-inflammatory factors IL-10 and TNF-β, and effectively inhibit osteoclast formation through the type I receptor made up of common gamma chain (γC) subunits and IL-4Rα. IL-4 deficiency can lead to more severe cartilage injury and osteophyte formation [89]. Based on the anti-inflammatory effect of M2 macrophages, researchers have demonstrated that artificial M2 macrophages, which are composed of the macrophage cell membrane as the “shell” and the inflammation-responsive gel as the “yolk,” can downregulate the inflammatory response during the acute reaction period and can continuously release chondroitin sulfate and other substances in the gel during the low inflammatory activity period to achieve continuous repair of cartilage [89]. Other studies further confirmed that M2 macrophages play an anti-inflammatory role in the development of primary OA disease [89, 90].

**Table 1:** Summary of treatment of osteoarthritis associated with macrophage polarization

Name	Study model	Immunoregulatory function	Signaling pathways	Ref.
Metformin	DMM-induced OA mouse model; BMDMs; RAW264.7 cells	M1↓	mTOR	[51]
EVs	bone marrow mesenchymal stem cell-derived exosomes; RAW264.7 cells	M1↓ M2↑	mTOR	[56]
Clodronate	C57 mice received a closed articular fracture	M1↑ M2↓	None	[64]
Triamcinolone	Primary human monocytes	M1↓ M2↑	mTOR	[66]
Alpha defensin-1	THP-1 human monocytic cell line; chondrocytes of OA patients	M1↓ M2↑	Insulin signaling and Toll-like receptor pathways	[29]
Dexamethasone	ACLT-induced OA rat and rabbit models	M1↓ M2↑	mTOR	[65]
Apigenin	Hulth surgery induced OA mouse	M1↓ M2↑	mTOR/MAPK	[76]
SHP099	DMM-induced OA mouse model; RAW264.7 cells	M1↓	TLR/MyD88	[77]
Nrf2	NA	M1↓ M2↑	TGF-β/SMAD、TLR/NF-κB and JAK/STAT	[69]

## Discussion

Macrophages, as one of the immune cells, play a significant part in the induction and maintenance of chronic inflammation in primary OA. Current evidence suggests that OA synovial inflammation is dependent on the proliferation and activation of macrophages. Activated M1 macrophages can accumulate in large quantities in the synovial membrane and IPFP, produce a variety of proinflammatory factors and MMPs through the paracrine mechanism, and communicate with chondrocytes and adipose tissues. This process promotes balancing the synthesis and degradation of the ECM maintained by chondrocytes through catabolism, leading to cartilage injury and osteophyte formation; it further stimulates macrophage activation, promotes M1/M2 macrophage imbalance, and aggravates synovial inflammation and cartilage injury. M1 affects bone metabolism and accelerates subchondral bone remodeling by promoting osteoclast maturation. The targeted reduction in the number of macrophages slowed down primary OA pain; however, reduction in the number of cells did not delay disease progression. Further experiments confirmed that the inhibition of M1 polarization or enhancement of M2 polarization could effectively alleviate primary OA disease progression. Thus, regulating the polarization of M1 and M2 macrophages or promoting the transformation of M1 macrophages to M2 macrophages is a more effective approach to prevent and treat primary OA disease, rather than solely reducing the number of macrophages. Sadly, the preclinical stage is the only one covered by these investigations. Another limitation is that dividing macrophages into M1 or M2 types only on the basis of their functions in physiology and the development of illness is no longer sufficient because macrophages are a highly heterogeneous and plastic cell subset. Therefore, cell surface markers are often used to classify M1 and M2 kinds of macrophages. Research has revealed that certain subtypes of macrophages, which are more numerous than M1 and M2 macrophages, contribute significantly to the development of primary OA [19]. It is unclear, nevertheless, which subtypes of macrophages with particular functions can serve as the most effective targets for primary OA. In addition, the immune activation mechanism of the anti-inflammatory effect of tissue-resident macrophages in the knee joint and the pro-inflammatory effect of MDM are still unclear. Single-cell RNA sequencing and immunohistochemistry will continue to be required in the future to tease out the spatial and temporal characteristics of macrophages derived from diverse sources. It is undeniable that controlling macrophage polarization helps prevent primary OA from progressing and promote the transformation of M1 macrophages into M2 macrophages, which helps to lessen pain, preserve cartilage, lessen synovial inflammation, and interrupt the malignant cycle that leads to primary OA. Following further studies on primary OA, this disease is gradually classified into various subtypes, which poses a new challenge to study the mechanism of action of macrophages after polarization.

This review summarizes the biological relationship between macrophages and the surrounding cells and briefly analyzes the mechanism of action. Several in vitro experiments confirmed the feasibility of targeted regulation of macrophage polarization for preventing and treating primary OA disease. Further studies should investigate the fine tuning of macrophage polarization in OA.

## Data Availability

No datasets were generated or analysed during the current study.

## Abbreviations

OA: osteoarthritis
IAHA: intraarticular hyaluronic acid
PRP: platelet-rich plasma
IFN-γ: interferon-γ
TNF-α: tumor necrosis factor-α
LPS: lipopolysaccharide
MDMS: monocyte-derived macrophages
DAMPs: damage-associated molecular patterns
TLR: Toll-like receptor
IRFs: interferon regulatory factors
NF-kB: nuclear factors
MMP: matrix metalloproteinase
NLRP3: Pyrin domain 3
EVs: extracellular vesicles
METTL3: methyltransferase-like protein 3
IPFP: infrapatellar fat pad
VEGF: vascular endothelial growth factor
HIF-1α: hypoxia-induced-factor-1α
PlGF-1: placental growth factor 1
MCP-1: monocyte chemoattractant protein 1
ECM: extracellular matrix
ACAN: aggrecan
COL2: type II collagen
ADAMTS4: agglutinase 1(a disintegrin and metalloproteinase with thrombospondin motifs–4)
ADAMTS5: agglutinase 2
MPCs: mesenchymal progenitor cells
Acs: apoptotic cells
GAS6: growth arrest protein 6
FR-β: folate receptor-β
SPECT-CT: single photon emission computed tomography combined with high resolution computed tomography
Fpr1: formyl peptide receptor 1
MSCs: marrow-derived stem cells
DRG: dorsal root ganglion
PRRs: pattern recognition receptors
PAMPs: pathogen-associated molecular patterns
BCP: basic calcium phosphate
CPPD: calcium pyrophosphate dihydrate
IRAK4: interleukin-1 receptor-associated kinase 4
TRAF6: tumor necrosis factor receptor-associated factor 6
TAK-1: TGF-beta-activating kinase 1
IKK: IκB kinase
IκBα: NF-κB inhibitor
RIPK1: recruiting receptor-interacting protein kinase 1
IRF5: interferon regulatory factor 5
CS: chondroitin sulfate
Nrf2: Nuclear factor-erythrocyte 2-associated factor-2
TLRS: Toll-like receptors
IL-1β: interleukin-1β
OPG: osteoprotegerin
RANKL: NF-κB receptor activator ligand
RANK: NF-κB receptor activator
FLS: fibroblast-like synovial cells
VEGF: vascular endothelial growth factor
γC: gamma chain
